# Supplementary figures and images for: Sensorimotor integration enhances temperature stimulus processing
Source: PLoS Comput Biol. 2025 Jun 10;21(6):e1013134. doi: 10.1371/journal.pcbi.1013134 (PMC12151342; doi:10.1371/journal.pcbi.1013134)

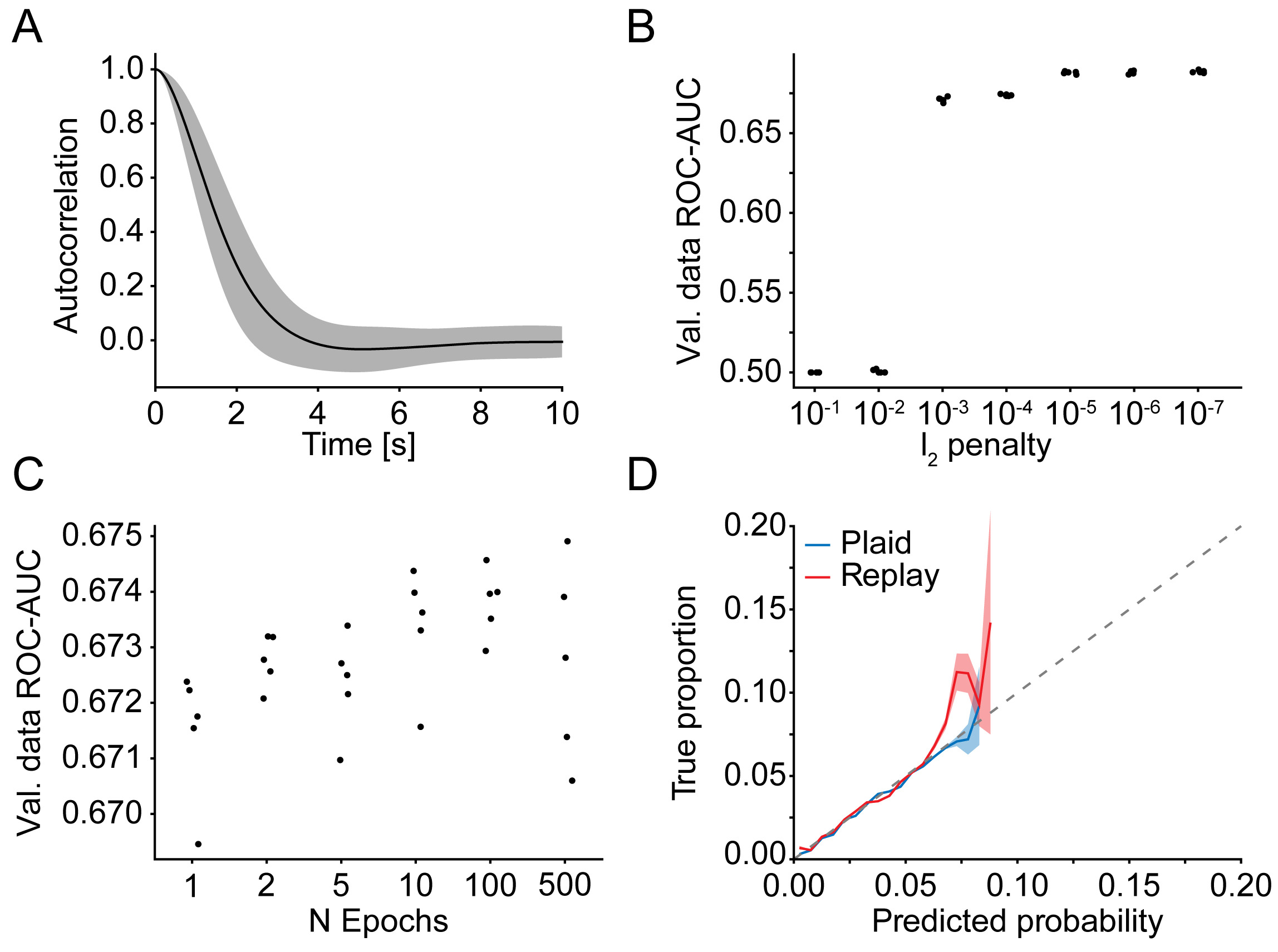

Supplement: S1 Fig [file pcbi.1013134.s001.tif]

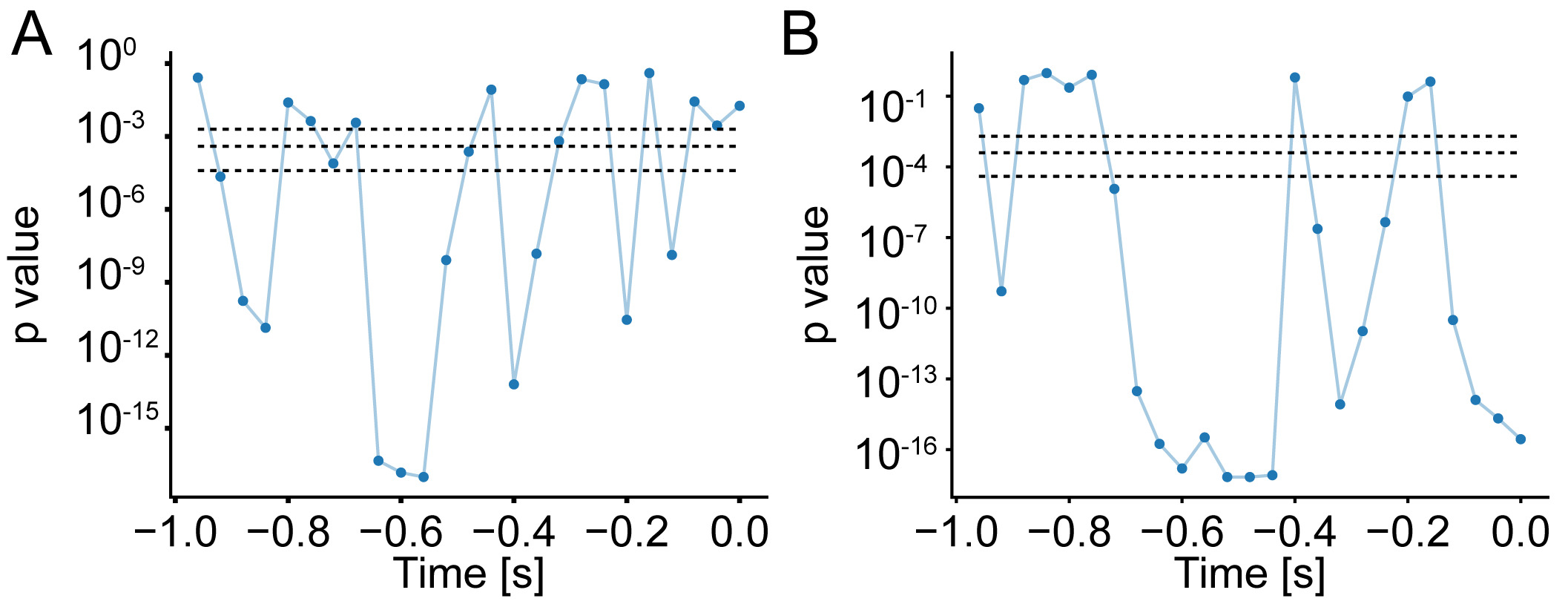

Supplement: S2 Fig — A) Blue dots: For each time point the p-value obtained from a ranksum test comparing the temperature receptive field values of the Plaid and Replay condition (N = 100 model fits). The black lines show p < 0.05; p < 0.01; p < 0.001 significance level after correcting for multiple comparison across 25 time points. B) Blue dots: For each time point the p-value obtained from a ranksum test comparing the bout history receptive field values of the Plaid and Replay condition (N = 100 model fits). The black lines show < 0.05; p < 0.01; p < 0.001 significance level after correcting for multiple comparison across 25 time points. (TIF) [file pcbi.1013134.s002.tif]
