# Supplementary material for: Sensorimotor integration enhances temperature stimulus processing
Source: PLoS Comput Biol. 2025 Jun 10;21(6):e1013134. doi: 10.1371/journal.pcbi.1013134 (PMC12151342; doi:10.1371/journal.pcbi.1013134)
Supplement: Attachment — Submitted filename: Review of Anderson et al 2025.pdf [file pcbi.1013134.s004.pdf]

**Comment on prior editorial rejection:**

In prior work (ref. 12 in the current manuscript), the Haesemeyer lab developed a method using convolutional neural nets (CNNs) to extract neural receptive fields. Though that paper thoroughly demonstrated the utility of this technique (called "model identification of neural encoding, or "MINE") in exploring multiple datasets, including one from zebrafish performing thermally-induced behavior, the method is applied here to a novel behavioral paradigm, in service of a unique set of questions. Particularly given the importance of the central findings of this work, I feel the paper more than meets the standards for careful consideration by this journal.

**Summary of this study:**

This current manuscript uses MINE and a new behavioral paradigm to investigate an important question: How do sensory feedback and the activity of so-called "mixed-selectivity" neurons – i.e. those that respond jointly during sensory and motor events – affect behavior?

The question is motivated by prior behavioral and neurophysiological observations.

First, though it is known that fish can maintain temperature homeostasis through behavioral means – i.e. by using an "active sensing" process to migrate to regions of higher or lower temperature – environmental conditions do not always provide the relatively sharp thermal gradients needed to accomplish this. Under such conditions, the animal's movement will repeatedly fail to produce a change in body temperature. Does the animal adopt different behavioral strategies depending on whether its behavior is effective or ineffective?

Second, previous work from the Haesemeyer lab used whole-brain calcium imaging to identify neurons that selectively encode thermal stimuli, locomotion, or a combination of both quantities. The features of the latter, "mixed-selectivity" neurons are in principle appropriate for evaluating behavioral effectiveness and changing the navigation strategy employed. Are they in fact used for such a purpose?

The paper uses a new behavioral paradigm in combination with extensive modeling to argue convincingly that the presence of sensory feedback indicating behavioral effectiveness increases the dependence of behavior on prior sensory experience. Working with CNN models describing mixed-selectivity neurons that were identified in the Costabile et al. 2023, it then shows that such neurons provide a powerful means of representing sensorimotor feedback and encoding the appropriate behavioral strategy.

**Positive features:**

The paper's greatest strength is the significance of its central findings. The demonstration that fish choose different navigation strategies depending on sensory feedback, and the finding that the integrated representation of sensory experience and behavior provided by mixed-selectivity neurons is optimal for encoding that strategy, are both highly novel.

The persistence of swimming behavior under "replay" conditions also provides an interesting and important contrast to other examples of behavior under futile conditions, specifically the case of behavioral passivity that has been observed by the Ahrens lab when measuring optomotor behaviors in an open-loop configuration (Mu et al. 2019). The behavioral difference across sensory modalities seems to reflect quite different ethological significances for vision versus temperature in a natural context.

The methodological approach is also quite strong throughout. This includes the novel use of the "thermal plaid" environment to provide sensory feedback to free-swimming fish, as well as its comparison to unstructured yoke controls. These are technically challenging and conceptually novel experiments.

The application of the previously developed method for extracting sensory and behavioral receptive fields using a CNN-based approach to this behavioral paradigm is conceptually novel and highly appropriate.

The project's rationale, significance, and experimental approach are uniformly excellent.

**Negatives:**

Statistical tests are lacking in a few key places:

- Figure 2a-c: The behavioral distributions of course seem to be highly similar. However, these data need some sort of statistical metric to formalize this assertion.
- Figure 3c-f: Same problem. Controls were generated by rotating the CNN outputs relative to its inputs, which is appropriate. There are qualitatively compelling differences between the RF coefficients derived from plaid vs. replay conditions, and in comparison to the rotated controls. However, I would like to see a statistical estimate of the likelihood of each difference arising by chance.

Fig 4 uses CNN models derived to predict mixed-selectivity neuron activity elicited by different sensorimotor histories. The paper these models come from (Costabile et al. 2023; ref 12) performed the calcium imaging experiments needed for their derivation in head-fixed animals in an open-loop behavioral paradigm. Is it valid to use CNNs derived in this way on data derived from free-swimming animals, and in the "plaid" condition, animals that were behaving in a closed-loop paradigm? If this is not a true concern, it should be justified in the text.

Also in Figure 4, a network of 1023 mixed-selectivity neurons is used. Why? Does this approximate the complement of such cells identified in a representative animal? This should be justified in the text.
