# Supplementary material for: Sensorimotor integration enhances temperature stimulus processing
Source: PLoS Comput Biol. 2025 Jun 10;21(6):e1013134. doi: 10.1371/journal.pcbi.1013134 (PMC12151342; doi:10.1371/journal.pcbi.1013134)
Supplement: Attachment — Submitted filename: Response_To_Academic_Editor_241205.docx [file pcbi.1013134.s003.docx]

PCOMPBIOL-D-24-01934
Sensorimotor integration enhances temperature stimulus processing
PLOS Computational Biology

Dear Dr. Haesemeyer,

Thank you for submitting your manuscript to PLOS Computational Biology. As with all papers, your manuscript was reviewed by members of the editorial board. Based on our assessment, we have decided that the work does not meet our criteria for publication and will therefore be rejected.

Your analysis of thermoregulatory behaviour is very interesting. However the computational aspect has already been presented in your previous publication Costabile, J. D., Balakrishnan, K. A., Schwinn, S. &
Haesemeyer, M. 2023 (n.b. the bibliography is missing the journal and year references), and thus the current manuscript does not meet the journal's criteria for novelty.

We thank the academic editor for finding our analysis “very interesting.” However, we have difficulty understanding the reason for the rejection. It is correct that the method we apply in this paper has been published by us in a previous paper. However, “the computational aspect” we focus on in this manuscript is novel. Specifically, we apply the technique we developed to new behavioral data in order to compare computational strategies larval zebrafish use to thermoregulate across two conditions: One in which the animal receives thermosensory feedback during movements and the other where it passively experiences the same stimulus. This allows us to address a novel question: Do animals adjust how they process temperature stimuli based on sensorimotor feedback.

The referenced ELife paper introduces the technique and in it we identified neurons that have a mixed encoding of temperature and behavior. In the current paper we apply this technique and further implicate the mixed selectivity neurons in the observed changes in thermosensory processing. Rather than “not meet[ing] the journal’s criteria for novelty” we would argue that this is standard scientific progress: Applying a published technique to a relevant question to obtain new insight into a computational biological question. We would furthermore argue that many papers published in PLOS Computational biology follow a similar approach. Applying well-established techniques to answer interesting scientific questions.

I apologize for the oversight in compiling the paper references. I used the wrong bibtex format when generating the PDF and omitted carefully checking the bibliography. We addressed this oversight in the resubmission.

We are sorry that we cannot be more positive on this occasion. We very much appreciate your wish to present your work in one of PLOS's Open Access publications. Thank you for your support, and we hope that you will consider PLOS Computational Biology for other submissions in the future.

We thank the editors for reconsidering their decision since we firmly believe that PLOS Computational Biology is the perfect home for our manuscript.

Yours sincerely,

Lyle Graham
Section Editor
PLOS Computational Biology

Feilim Mac Gabhann
Editor-in-Chief
PLOS Computational Biology

Jason Papin
Editor-in-Chief
PLOS Computational Biology

**Additional Editor Comments (if provided):**


[Note: HTML markup is below. Please do not edit.]

**Reviewers' Comments (if peer reviewed):**


[NOTE: If reviewer comments were submitted as an attachment file, they will be attached to this email and accessible via the submission site. Please log into your account, locate the manuscript record, and check for the action link "View Attachments". If this link does not appear, there are no attachment files.]
