# Supplementary material for: Sensorimotor integration enhances temperature stimulus processing
Source: PLoS Comput Biol. 2025 Jun 10;21(6):e1013134. doi: 10.1371/journal.pcbi.1013134 (PMC12151342; doi:10.1371/journal.pcbi.1013134)
Supplement: Attachment — Submitted filename: Response_to_reviewers.docx [file pcbi.1013134.s005.docx]

We thank the reviewers for engaging with our manuscript and for their insightful comments. We have addressed the issues that were raised and believe that this strengthened our manuscript. Our answers to specific points brought up by the reviewers are in blue below.

**Reviewer 1**

**Summary of this study:**

This current manuscript uses MINE and a new behavioral paradigm to investigate an important question: How do sensory feedback and the activity of so-called “mixed-selectivity” neurons – i.e. those that respond jointly during sensory and motor events – affect behavior?

The question is motivated by prior behavioral and neurophysiological observations.

First, though it is known that fish can maintain temperature homeostasis through behavioral means – i.e. by using an “active sensing” process to migrate to regions of higher or lower temperature – environmental conditions do not always provide the relatively sharp thermal gradients needed to accomplish this. Under such conditions, the animal’s movement will repeatedly fail to produce a change in body temperature. Does the animal adopt different behavioral strategies depending on whether its behavior is effective or ineffective?

Second, previous work from the Haesemeyer lab used whole-brain calcium imaging to identify neurons that selectively encode thermal stimuli, locomotion, or a combination of both quantities. The features of the latter, “mixed-selectivity” neurons are in principle appropriate for evaluating behavioral effectiveness and changing the navigation strategy employed. Are they in fact used for such a purpose?

The paper uses a new behavioral paradigm in combination with extensive modeling to argue convincingly that the presence of sensory feedback indicating behavioral effectiveness increases the dependence of behavior on prior sensory experience. Working with CNN models describing mixed-selectivity neurons that were identified in the Costabile et al. 2023, it then shows that such neurons provide a powerful means of representing sensorimotor feedback and encoding the appropriate behavioral strategy.

**Positive features:**

The paper’s greatest strength is the significance of its central findings. The demonstration that fish choose different navigation strategies depending on sensory feedback, and the finding that the integrated representation of sensory experience and behavior provided by mixed-selectivity neurons is optimal for encoding that strategy, are both highly novel.

The persistence of swimming behavior under “replay” conditions also provides an interesting and important contrast to other examples of behavior under futile conditions, specifically the case of behavioral passivity that has been observed by the Ahrens lab when measuring optomotor behaviors in an open-loop configuration (Mu et al. 2019). The behavioral difference across sensory modalities seems to reflect quite different ethological significances for vision versus temperature in a natural context.

The methodological approach is also quite strong throughout. This includes the novel use of the “thermal plaid” environment to provide sensory feedback to free-swimming fish, as well as its comparison to unstructured yoke controls. These are technically challenging and conceptually novel experiments.

The application of the previously developed method for extracting sensory and behavioral receptive fields using a CNN-based approach to this behavioral paradigm is conceptually novel and highly appropriate.

The project’s rationale, significance, and experimental approach are uniformly excellent.

We are happy that the reviewer found our paper to be of high significance.

**Negatives:**

Statistical tests are lacking in a few key places:

Figure 2a-c: The behavioral distributions of course seem to be highly similar. However, these data need some sort of statistical metric to formalize this assertion.

We fully agree with the reviewer and we have made the following adjustments to 2a-c: We have replaced the histogram overlays with clearer line-representations with bootstrapped standard errors. These reveal pointwise differences. We have also added a bootstrapped ks-test for significant differences across the distributions which did not reveal significant differences between conditions. We note that we have chosen to bootstrap the ks statistic, since most variability arises between fish rather than individual swim-bouts. We therefore reasoned that a ks-test across the entire distribution of bouts would not be appropriate. For full disclosure, such a test shows high significance (p << 0.001) however, that is also the case across random subsets of fish. As outlined in a response to reviewer 3, we have also added baseline data to these plots.

Figure 3c-f: Same problem. Controls were generated by rotating the CNN outputs relative to its inputs, which is appropriate. There are qualitatively compelling differences between the RF coefficients derived from plaid vs. replay conditions, and in comparison to the rotated controls. However, I would like to see a statistical estimate of the likelihood of each difference arising by chance.

We agree with the reviewer that it is important to formalize the differences through a statistical test. Instead of adding annotations to the graphs we opted to include a new Figure S2, which for each timepoint shows the p-value of a ranksum test comparing the receptive fields for Plaid and Replay conditions. We also drew three standard significance levels (0.05, 0.01, 0.001) into these graphs after correcting for multiple comparisons across 25 timepoints. We did not include similar plots for each condition versus the rotated controls for simplicity as the p-values for the Plaid-Replay condition track the presence or lack of overlap of the standard errors plotted in 3c-f very well.

Fig 4 uses CNN models derived to predict mixed-selectivity neuron activity elicited by different sensorimotor histories. The paper these models come from (Costabile et al. 2023; ref 12) performed the calcium imaging experiments needed for their derivation in head-fixed animals in an open-loop behavioral paradigm. Is it valid to use CNNs derived in this way on data derived from free-swimming animals, and in the “plaid” condition, animals that were behaving in a closed-loop paradigm? If this is not a true concern, it should be justified in the text.

We agree with the reviewer that this is a valid concern. In the main text we therefore lay out our reasoning for why we believe the models to be an adequate approximation. We state: “*These models were fit under open-loop conditions (akin to the Replay condition). Therefore, in the context of the closed-loop Plaid condition, naturalistic neural representations may not be perfectly recapitulated. However, analysis of these neurons suggested that their responses depend on the coincidence of temperature change and behavioral responses, which occurred during the imaging paradigm. This suggests that the responses we identified approximate the true physiological tuning of these neurons which can therefore be replicated by MINE.*”

Also in Figure 4, a network of 1023 mixed-selectivity neurons is used. Why? Does this approximate the complement of such cells identified in a representative animal? This should be justified in the text.

We agree with the reviewer and better justify our choice in the text. We state “*In Costabile et al., 2023, we found that neuronal subtypes were broadly represented in all animals. Consequently, we performed PCA on the complete set of 1023 neurons to reflect the average activity space characteristic of all fish, rather than the precise neuronal composition of any single individual.*”

**Reviewer 2**

The authors of 'Sensorimotor integration enhances temperature stimulus processing' investigate if behavioral feedback coupled to changes in sensory input allow for more effective temperature control. Using a behavioral paradigm, they first demonstrate that an animal that navigates in a spatial environment with a structured temperature pattern is more effective at reducing its temperature experience, compared to an animal where movement and stimulus are uncoupled. I overall found this paper a compact, well-described observation with the computational aspects aiding to suggest potential mechanisms for the observation. However, I have a few general comments regarding a few statements and comparisions that if adressed would strengthen the results.

The effect size of Fig. 1 E seems rather small. It would be useful to put this result in context - what are the changes in temperature across the 'plaid' and what percentage does the 0.1 degree Celsius represent? (This comes later in the 'Limitations') but it would help the reader to have context here.

We agree with the reviewer and have now added information to the first results section. Here, we state the amplitude of the Plaid as well as its spatial extent. We also compare the experienced changes in temperature to those that drive behavioral changes within gradients and state: “*Although these changes are small, larval zebrafish use differences on the order of 0.1 C to modulate behavior during gradient navigation (Palieri, 2024).*”

In Figure 2, the authors suggest that the overall behavior in stimulus-coupled and uncoupled animals is similar. They begin their argument with learned helplessness, an effect previously described for visual paradigms. They then dismiss this by showing that gross behavioral statistics are similar. Here, proof by graphical overlay is used, which in itself is not bad, however, I'm suprised the peak in Fig. 2A is not significant. Please run an appropriate statistic (for example a KS-test). The graphing of this information would benefit from either using linegraphs, or opacity to show both histograms more clearly.

We fully agree with the reviewer and we have made the following adjustments to 2a-c: We have replaced the histogram overlays with clearer line-representations with bootstrapped standard errors. These reveal pointwise differences. We have also added a bootstrapped ks-test for significant differences across the distributions which did not reveal significant differences between conditions. We note that we have chosen to bootstrap the ks statistic, since most variability arises between fish rather than individual swim-bouts. We therefore reasoned that a ks-test across the entire distribution of bouts would not be appropriate. For full disclosure, such a test shows high significance (p << 0.001) however, that is also the case across random subsets of fish within the same condition. As outlined in a response to reviewer 3, we have also added baseline data to these plots which we now use as a clearer argument against learned helplessness.

In addition, I wonder if learned helplessness would not show up over time, rather than in overall statistics. However, this is in general a side-note and I don't think this aspect is central to the paper, and I was generally a bit distracted by this Figure. In general, the latter part of the paper argues that swim bouts are different (e.g. Fig. 3E) so this line of reasoning should be clarified in the text.

The comparison to baseline data makes a clearer argument against learned helplessness. Under both conditions the stimulus leads to an enhancement in overall swim vigor. While this cannot rule out that learned helplessness would develop over longer timescales, it seems less likely. We also believe that our change to line graphs as suggested by the reviewer helps to clear up or argument surround the investigation of receptive fields. We thank the reviewer for this insight.

The authors then use a previously published CNN-based approach to determine receptive fields from non-white noise (i.e., highly correlated) stimulus data. The paper would be easier to read if the authors added a (minimal) description of this model is and how it works. White-noise based approaches are widespread, as they have some neat statistical properties. It would be useful to state why this alternate approach works.

We agree with the reviewer and have added the following to introduce the approach: “Since white-noise stimuli are random and by construction controlled by the experimenter rather than the animal, we could not use them for our current purpose since they would lack sensorimotor feedback. In the current experiments, were larval zebrafish controlled the temperature stimulus, we instead faced the challenge of long stimulus autocorrelation times (Figure S1) which makes it challenging to extract receptive fields. We therefore used a method based on a convolutional neural network (CNN) we recently developed, Model Identification of Neural Encoding (MINE) (Costabile , 2023). This approach allows to recover receptive fields even for stimuli with high autocorrelation times, by fitting a CNN relating the stimulus to the response and subsequently using Taylor expansion to extract the receptive field (Costabile, 2023). We previously demonstrated that this approach requires less tuning than receptive field computations based on regularized linear regression and generally yields a more faithful representation of the true receptive field (Costabile, 2023).” We hope that this gets to the reviewer’s point.

The final section concerns inference of mixed selectivity neurons on behavior, with the rationale that these neurons may encode relevant information such that it is easily decodable later on. As this paper is using only behavioral data, a previously used generative model supplied neuronal traces which were then fed into a classifier to predict bouts. This model is compared to a linear classifier and performs better. However, this comparison is flawed: The neuron model has access to non-linear features extracted from the data, wheras the linear classifier is restricted to linear features. It is very obvious that this would be a mismatched comparison. A better comparison could be a non-linear classifier, a random forrest or at least a random weight CNN with similar layers.

It seems that our writing obscured the intent of the comparison. The whole point we aim to make is that the neural circuits, represented by the CNN models, are transforming the information such that linear decoding will be possible. We therefore use a linear classifier both on the neural data and the input data to ask whether the neural data represents the information in way that is easier to decode than the inputs themselves. So, our results suggest that the nonlinearity of the neuron model, which is dictated by the nature of the responses of mixed selectivity neurons in the fish, is aiding in decoding the conditions. We have edited this section to hopefully make our intent clearer.

Specific line edits:

Ln 54: This sentence sounds like editorializing and is currently not supported by references. I suggest reformulating and providing evidence.

We agree with the reviewer and now state instead: “*When larval zebrafish are deprived of visual feedback during behavior, they strongly suppress motor output, entering a state of learned helplessness (Mu, 2019). A reason for this might be that when animals move through the world, they generally experience optic flow (Koenderink, 1986) and e.g., rodents will compute mismatch signals when this expectation is violated (Keller, 2012; Keller, 2018). The complete absence of visual feedback may therefore signal that behavior is futile since it appears to the animal as if it is not moving.*”

Fig. 3B, caption: 'rotated control data' - do you mean circularly permuted? Shifted? Or indeed a spatial rotation relative to the plaid? This phrase is otherwise unfamiliar to me.

We excuse the colloquialism. We now state “*circularly permuted*”. We also realized that we did not define this control in the method section. We now added a definition of the circular permutation to the “Network model and training” section.

Minor: Figure 1:- formatting of superscripts in the caption

We thank the reviewer for spotting this. Throughout the paper we have decided to report all p-values smaller than 0.001 as < 0.001 instead of the exact value to avoid suggesting an unrealistic level of significance.

Figure 3B: formatting of p-value and statistics-

We fixed the erroneous math-mode formatting in the legend.

Ln.151 Are actively sought -> is actively sought

We thank the reviewer for spotting this and have fixed the error.

**Reviewer 3**

Overall I like the paper -- it's fun to read and interesting, and gets at some important issues in behavioral neuroscience about how information is incorporated by animals when making behavioral decisions. I would appreciate some aspects being discussed a bit more systematically, and a have a basic question about the framing of the main result, but otherwise the comments are relatively minor.

My broader comment is about framing. The fish are described as "actively controlling" the temperature because there is a direct connection between where they move and what temperature stimulus they experience. To me this seems like the default in a ecological setting, and would also be true for terrestrial animals moving around on a spatial thermal gradient, for example. Unless the fish are making spatial maps and storing that information in their brain (are they? I don't recall it coming up), wouldn't ANY movement appear TO THE FISH as the same thing, regardless of how temperature was changing with time? I understand that in Replay conditions the fish are not moving in a virtual plaid gradient anymore, but they do still have the temperature change as they move. It seems like the main difference between Plaid and Replay is what happens when the fish is sitting still -- in Replay they might experience temperature changes, but never in Plaid. Could another way to talk about the difference in the experiments is that in one type their stationary periods are being interrupted and in the other they are not? From the picture in Fig. 1C it seems like navigating that environment would provide plenty of sensory feedback even if kind of scrambled. I'm not asking to change how you talk about everything, but it might be useful to acknowledge what happens differently from the fish's point of view. For them every stimulus is temporal.

We agree with the reviewer that our framing of the two different conditions requires clarification. We fully agree, that this is not fish specific – any animal moving through a temperature gradient in nature will experience temperature changes locked to behavior. However, temperature can also change across time (differences in sun irradiation in the presence/absence of clouds) independent of movement. In which case there is no gradient the animal could exploit for thermoregulation. To better clarify what happens from the fish’s perspective we now say in the first results section: “*In the Replay condition on the other hand changes in temperature were uncoupled from behavior (Figure 1D). From the fish’s perspective, the largest change in temperature should occur while moving (i.e., during swim bouts) in the Plaid condition, while temperature changes should be evenly distributed across bout and interbout periods in the Replay condition. This is indeed what we found.*”

And in the Introduction we now state: “*However, it is unknown whether animals integrate information about behavioral actions with thermosensory feedback to optimize thermoregulatory behavior; e.g., coincidence of movements and perceived changes in temperature could inform animals about the presence of thermal gradients, while integrating information about travel distance with temperature change could be used to infer the gradient's slope. Absence of a link between changes in temperature and movement would on the other hand signal environmental factors such as changes in cloud cover which the animal cannot exploit for thermoregulation. Here, we address whether animals use thermosensory feedback of behavior in the vertebrate model larval zebrafish.*”

It seems here that the main result of the paper is that when the temperature changes during bouts, the fish pay behavior is more influenced/controlled by the stimulus. If that's true, wouldn't it not be very important to have any kind of coherent spatial pattern? If you change the temperature every time the fish moved, would you expect similar results? Or similarly, intentionally giving fish changing temperatures while they are at rest but NOT when they are in bouts would minimize the relationship between behavior and stimulus.

We agree with the reviewer, that the important cue for zebrafish is the link of temperature changes to swim bouts, not the spatial pattern and the experiments suggested by the reviewer would likely produce the expected results. The plaid stimulus is a convenient way to enforce a link between behavior and temperature change. Since swim bouts only last on the order of 100-200 ms, it would be challenging to perform the experiments the reviewer suggests. Ad-hoc bout detection to induce a change in laser power is more error prone than having the position determine the power. We have added the following to the end of the discussion to emphasize the point made by the reviewer: *“These findings suggest that larval zebrafish infer the presence of a gradient from the relationship of temporal temperature dynamics with behavior rather than specific spatial patterns. This mirrors natural ecology where fish infer spatial thermal gradients through temporal sequences generated by navigation (Haesemeyer, 2020) effectively using time to reconstruct space.”*

The Introduction is really short, and most of it isn't background, but it's the abstract material repeated and then talking about the experiments in the manuscript itself. Setting a broader context would be helpful I think. Actually I think the examples in line 155ff and 183ff would fit nicely in the Introduction.

We agree with the reviewer and have expanded the introduction.

What percentage of their time do the fish spend in bouts? Are they traces in Fig. 1D fairly representative, or are they unusual because of the changing temperature stimulus? It would be helpful to have a baseline idea.

We fully agree with the reviewer and we now give a better idea about the structure of swim bouts in the first part of the results where we state: “*Larval zebrafish swim in discrete bouts that are on average 100-200 ms long. These swim bouts are separated by stationary periods, the interbout intervals which last 500-1800 ms. We therefore focused our paradigm on modulating the amount of sensory feedback larval zebrafish would receive during the swim periods versus the intervening interbout periods.*”

The apparatus is very cool, but I think it would help if the reader understood better why it was important/necessary to build it. For example, for a land-crawling creature a fixed spatial gradient could accomplish the same thing as Plaid -- is being in water what would make that difficult?

We agree with the reviewer that this is very helpful information. We now added the following to the first section of the results: “*This setup allowed us to present thermal gradients as a fish might experience in nature by modulating temperature according to fish position, as well as presenting changes in temperature that are decoupled from position which would be difficult to obtain in a gradient setup. Using this setup, we modulated the temperature of larval zebrafish according to one of two rules (Figure 1B). In the first case, temperature was coupled to the position of the fish within a 10 cm sized arena in the form of a ``thermal plaid'', forming multiple mini-gradients ranging from 28 C at the warmest point to 25 C at the coolest over a distance of 21 mm (``Plaid'' condition). We chose a plaid-like temperature profile for two reasons. Within the plaid, fish will experience both increases and decreases in temperature even for short movement distances and unlike a circular gradient, there is no defined relationship between temperature and distance to the edge of the dish which avoids confounds introduced by larval zebrafish hugging the edge of the arena (thigmotaxis (Schnorr, 2012)).*”

(~line 60) There is a clear comparison with Plaid/Replay -- could there be some comment or data shown what the fish's baseline behavior is like in an isotropic environment as well?

We fully agree with the reviewer that a baseline comparison is very useful. We added this data to Figure 2 and discuss it in the relevant result section.

(line 107) I couldn't tell what precisely delta_f means. It says changes in bout frequency -- does this mean between subsequent bouts? Or before vs. after the stimulus shows up? And why does having a wider distribution of this quantity mean there is a stronger influence from the stimulus on behavior? This might be addressed in other work, but I feel I'm missing some of the logic in the manuscript text.

We agree with the reviewer that this section lacked clarity. We have now expanded the text to hopefully remedy this issue. We state: “*Since the receptive field measures how much the stimulus affects the probability of swim bouts, we used the Plaid and Replay receptive fields to compute the swim probability at each time point in each experiment. This allowed us to determine the change from the average bout frequency mediated by the thermosensory receptive field across time. As expected, the stimuli experienced by larval zebrafish during the experiments were predicted to have a stronger influence on behavior in the presence of sensorimotor feedback than in the Replay condition. This can be seen in the overall distribution of changes in bout frequency (Figure 3E). Here, a wider distribution signifies that the stimulus induces a larger range of bout frequencies between times where the stimulus is opposed to the receptive field and times where it is aligned with the receptive field in the Plaid compared to the Replay condition. This can also be seen in the per-experiment standard deviation of stimulus bout frequency modulation across time induced by the receptive fields (Figure 3F).*”

(line 109) Maybe rewrite this sentence for clarity, it's a little clunky.

We have rewritten the sentence to read.

(line ~285) The turn angle means the drift in direction during a bout? Why does it change so much during the bout (seen in Fig. 1D) -- and why does it have noisy bursts like that?

We have updated the legend for figure 1D to better describe what is seen in the panel. It now reads: “*Illustration of extracted swim bout kinematics and 5 second long example trace for a pair of Plaid (left) and Replay (right) experiments showing delivery of the same stimulus but change in sensorimotor feedback. Stimulus temperature top, swim speed in second row revealing structure of larval zebrafish movement as periods of rests with intermittent swim bouts, third row reveals tail movements that are used as sensitive indicators to delineate the starts of swim bouts, bottom row shows fish heading within the arena revealing turns and straight swims; swim periods are marked in purple. We note that the rapid alternations in heading during swim-bouts are caused by the tail beats of the fish. Inset in Plaid stimulus trace shows an enlarged view of the pattern depicted in C with the example movement depicted across D overlaid as a white line.”*

In Fig. 1, it would be really nice to see the Plaid pattern (and the other one) larger, and perhaps with a real trajectory overlaid? (either the one from 1D or another representative one). It would be a nice visual and would also give a better sense of the scale of bout distances compared to the gradient size.

We thank the reviewer for this suggestion, and have added the requested inset corresponding to the trajectory in 1D.

Reference [15] might have a mistake (volume 0?)

We thank the reviewer for spotting this and have corrected the mistake.

(line 184) I think most of those genus/species should be in italics.

This has been corrected.

(I'm not clear on what a more "structured" output is? The term is used a lot and I'm not sure of the precise meaning.

We fully agree with the reviewer that this isn’t a standard term. We now define what we mean in the Introduction where we say: “*Our analysis suggests that behavior becomes more predictable as its temporal structure becomes more coherent (i.e., structured output) and that the stimulus exerts a larger influence on behavior during sensorimotor feedback.*”

The limitations section is pretty light -- the whole study has only one limitation? Some more thought on what could be done to expand the scope or test things in additional ways would be appreciated. Measuring neural activity for example?

We agree with the reviewer that a more thorough discussion on limitations is warranted. We added a paragraph to the end of the section to address limitations in our approach to mixed selectivity neurons.
